# Stimulation of JNK Phosphorylation by the PTTH in Prothoracic Glands of the Silkworm, *Bombyx mori*

**DOI:** 10.3389/fphys.2018.00043

**Published:** 2018-02-05

**Authors:** Shi-Hong Gu, Gen Li, Hsiao-Yen Hsieh, Pei-Ling Lin, Sheng Li

**Affiliations:** ^1^Department of Biology, National Museum of Natural Science, Taichung, Taiwan; ^2^Graduate School of Engineering, Chiba University, Chiba, Japan; ^3^Guangzhou Key Laboratory of Insect Development Regulation and Application Research, Institute of Insect Sciences and School of Life Sciences, South China Normal University, Guangzhou, China

**Keywords:** PTTH, JNK, ERK, ecdysone, signaling, redox regulation

## Abstract

In this study, phosphorylation of c-Jun N-terminal kinase (JNK) by the prothoracicotropic hormone (PTTH) was investigated in prothoracic glands (PGs) of the silkworm, *Bombyx mori*. Results showed that JNK phosphorylation was stimulated by the PTTH in time- and dose-dependent manners. *In vitro* activation of JNK phosphorylation in PGs by the PTTH was also confirmed in an *in vivo* experiment, in which a PTTH injection greatly increased JNK phosphorylation in PGs of day-6 last instar larvae. JNK phosphorylation caused by PTTH stimulation was greatly inhibited by U73122, a potent and specific inhibitor of phospholipase C (PLC) and an increase in JNK phosphorylation was also detected when PGs were treated with agents (either A23187 or thapsigargin) that directly elevated the intracellular Ca^2+^ concentration, thereby indicating involvement of PLC and Ca^2+^. Pretreatment with an inhibitor (U0126) of mitogen-activated protein kinase (MAPK)/extracellular signal-regulated kinase (ERK) kinase (MEK) and an inhibitor (LY294002) of phosphoinositide 3-kinase (PI3K) failed to significantly inhibit PTTH-stimulated JNK phosphorylation, indicating that ERK and PI3K were not related to JNK. We further investigated the effect of modulation of the redox state on JNK phosphorylation. In the presence of either an antioxidant (N-acetylcysteine, NAC) or diphenylene iodonium (DPI), PTTH-stimulated JNK phosphorylation was blocked. The JNK kinase inhibitor, SP600125, markedly inhibited PTTH-stimulated JNK phosphorylation and ecdysteroid synthesis. The kinase assay of JNK in PGs confirmed its stimulation by PTTH and inhibition by SP600125. Moreover, PTTH treatment did not affect JNK or Jun mRNA expressions. Based on these findings, we concluded that PTTH stimulates JNK phosphorylation in Ca^2+^- and PLC-dependent manners and that the redox-regulated JNK signaling pathway is involved in PTTH-stimulated ecdysteroid synthesis in *B. mori* PGs.

## Introduction

Ecdysteroids regulate insect growth, molting, and metamorphosis; they are synthesized and secreted by the prothoracic glands (PGs) (Marchal et al., 2010; De Loof, 2011; Smith and Rybczynski, 2012; De Loof et al., 2013, 2015; Yamanaka et al., 2013). A neuropeptide, known as the prothoracicotropic hormone (PTTH) and produced by brain neurosecretory cells, activates ecdysteroidogenesis in PGs (Marchal et al., 2010; De Loof, 2011; Smith and Rybczynski, 2012; De Loof et al., 2013, 2015). Numerous studies were conducted to examine the complex PTTH signaling network. PTTH activates PGs by binding to its receptor, Torso, which is a receptor tyrosine kinase (Rewitz et al., 2009, 2013; Smith and Rybczynski, 2012). A complex signaling transduction network is activated downstream of PTTH receptor activation (Rewitz et al., 2009; Marchal et al., 2010; Smith and Rybczynski, 2012). This network includes a rapid increase in Ca^2+^ (Gu et al., 1998; Birkenbeil and Dedos, 2002; Fellner et al., 2005), cAMP generation (Smith et al., 1984, 1985; Gu et al., 1996), and activation of protein kinase A (PKA), phospholipase C (PLC), protein kinase C (PKC), p70S6 kinase (S6K), ribosomal protein S6, and tyrosine kinase (Song and Gilbert, 1995, 1997; Smith et al., 2003; Rybczynski and Gilbert, 2006; Lin and Gu, 2011). Our recent studies further indicated that reactive oxygen species (ROS) and phosphoinositide 3-kinase (PI3K)/adenosine 5′-monophosphate-activated protein kinase (AMPK)/target of rapamycin (TOR) signaling are involved in PTTH-stimulated ecdysteroidogenesis in *Bombyx mori* PGs (Gu et al., 2011, 2012, 2013; Hsieh et al., 2013, 2014).

Mitogen-activated protein kinase (MAPK) cascades transduce a variety of signals in eukaryotic cells in response to multiple extracellular stimuli (Roux and Blenis, 2004). Depending on the cell type, duration of the stimulus, and pathway, they mediate a range of cellular responses including proliferation, differentiation, development, inflammation, and apoptosis. The most thoroughly characterized subgroups of the MAPK family include extracellular signal-regulated kinases (ERKs), c-Jun N-terminal kinases/stress-activated protein kinases (JNKs/SAPKs), and the p38 family of kinases (Widmann et al., 1999; Wetzker and Böhmer, 2003). Activated MAPKs are translocated to nuclei, where they phosphorylate a variety of target transcription factors (Roux and Blenis, 2004; Krishna and Narang, 2008). In insects, ERK phosphorylation appears to be involved in PTTH-stimulated ecdysteroidogenesis in both *Manduca sexta* and *B. mori* (Rybczynski et al., 2001; Lin and Gu, 2007; Gu et al., 2010; Gu and Hsieh, 2015). However, it is not clear whether other MAPK family members are involved in PTTH-stimulated ecdysteroidogenesis.

JNKs are a member of the MAPK family of protein kinases (Ip and Davis, 1998; Lewis et al., 1998; Weston and Davis, 2002). Mammalian JNKs were described as SAPKs, since they are activated by a variety of cellular stresses, such as UV light, heat, hyperosmotic shock, ROS, antioxidants, protein synthesis inhibitors, and inflammatory cytokines (Davis, 2000). In addition, JNKs are also activated by various growth factors, including prolactin, epidermal growth factor (EGF), platelet-derived growth factor (PDGF), nerve growth factor (NGF), insulin, insulin-like growth factor, and ligands for some G protein-coupled receptors. Phosphorylated JNKs subsequently bind to the NH_2_-terminal activation domain of c-Jun on Ser-63 and Ser-73, resulting in mediation of gene expression regulation (Weston and Davis, 2002, 2007). Similar to mammalian cells, the JNK signaling pathway is also conserved in *Drosophila*. The *Drosophila* JNK pathway consists of *Drosophila* JNK or basket (DJNK) and JNK kinase Hep, which are respective homologs of JNK and upstream JNK kinases in mammals (Sluss et al., 1996). *Drosophila* JNK signaling appears to be involved in various developmental processes, such as dorsal and thorax closure, wing development, control of morphogenetic apoptosis, regulation of imaginal disc proliferation, wound healing, and regeneration (Stronach and Perrimon, 1999; Bogoyevitch and Kobe, 2006). Both the ERK- and JNK-dependent signaling pathways appear to contribute to *Bombyx* nucleopolyhedrovirus infection (Katsuma et al., 2007). More recently, we reported that JNK signaling together with other MAPK signaling pathways, which are rapidly induced by injury, are related to diapause termination in dechorionated *Bombyx* eggs (Gu and Chen, 2017).

In the present study, we investigated the involvement of JNK in PTTH-stimulated ecdysteroidogenesis by *B. mori* PGs. We demonstrated that JNK phosphorylation was stimulated by PTTH both *in vitro* and *in vivo*. The kinase assay of JNK in PGs confirmed its stimulation by PTTH. The JNK inhibitor, SP600125, markedly inhibited PTTH-stimulated JNK phosphorylation and ecdysteroid synthesis, indicating the involvement of JNK phosphorylation in PTTH-stimulated ecdysteroidogenesis.

## Materials and methods

### Animals

Larvae of an F1 racial hybrid, Guofu × Nongfong, of *B. mori* were reared on fresh mulberry leaves at 25°C under a 12-h light: 12-h dark photoperiod. Newly-ecdysed last instar larvae were collected and used for each experiment.

### Reagents

SP600125, N-acetylcysteine (NAC), and diphenylene iodonium (DPI) were purchased from Sigma-Aldrich (St. Louis, MO, USA). Grace's insect cell culture medium was purchased from Invitrogen (Carlsbad, CA, USA). A MAPK/ERK kinase (MEK) inhibitor (U0126), a PI3K inhibitor (LY294002), A23187, and thapsigargin were purchased from Calbiochem (San Diego, CA, USA). All other reagents used were of analytical grade. [23, 24-^3^H] Ecdysone was obtained from New England Nuclear (Boston, MA, USA). Recombinant *B. mori* PTTH (PTTH) was kindly provided by Dr. David R. O'Reilly; it was produced by infection of *Spodoptera frugiperda-*SF21 cells with the vWTPTTHM baculovirus as described previously (O'Reilly et al., 1995). The same PTTH as that previously reported (O'Reilly et al., 1995; Gu et al., 2010) was used in the present study. In the present study, extracellular fluid from cells infected with vWTPTTHM was used as the PTTH source, and it was diluted 500 times with Grace's medium. Each incubation (50 μl) contained about 0.15 ng of PTTH.

### *In vitro* incubation of PGs, radioimmunoassay (RIA) of ecdysteroids, and *in vivo* injection of the PTTH

*In vitro* incubation of PGs and the RIA of ecdysteroids followed protocols described in a previous study (Lin and Gu, 2007). To study the *in vivo* activation of JNK phosphorylation of PGs by the PTTH, day-6 last instar larvae were injected with 10 μl saline containing 0.3 μl of the original PTTH solution. Larvae injected with 10 μl saline only were used as controls.

### Antibodies

Anti-phospho-SAPK/JNK (Thr183/Tyr185) (#9251), anti-phospho-ERK (#9101), anti-phospho-4E-BP (#9459), anti-total ERK (#9102), and anti-α-tubulin (#2144) antibodies were purchased from Cell Signaling Technology (Beverly, MA, USA). The anti-JNK1/3 antibody (sc-474) was purchased from Santa Cruz Biotechnology (Santa Cruz, CA, USA). Horseradish peroxidase (HRP)-labeled anti-rabbit immunoglobulin G (IgG) was purchased from PerkinElmer Life Sciences (Boston, MA, USA).

### Western blot analysis

Sodium dodecylsulfate polyacrylamide gel electrophoresis (SDS-PAGE) and immunoblotting were performed as described previously (Lin and Gu, 2007; Gu et al., 2010). Briefly, PGs from day-6 last instar larvae were homogenized in lysis buffer (10 mM Tris and 0.1% Triton x100) at 4°C, and then boiled in an equal volume of SDS sample buffer for 4 min followed by centrifugation at 15,800 *g* for 3 min to remove any particulate matter. One percent protease inhibitor cocktail (Sigma-Aldrich, cat. no. P8340) and 1% phosphatase inhibitor cocktail (Calbiochem, cat. no. 524629) were added at the time of homogenization. Aliquots of the supernatants were loaded onto SDS gels. Following electrophoresis, proteins were transferred to polyvinylidene difluoride (PVDF) membranes using an Owl (Portsmouth, NH, USA) Bandit™ Tank Electroblotting System, and then washed with Tris-buffered saline (TBS) for 5 min at room temperature. Blots were blocked at room temperature for 1 h in TBS containing 0.1% Tween 20 (TBST) and 5% (w/v) nonfat powdered dry milk, followed by washing three times for 5 min each with TBST. Blots were incubated overnight at 4°C with primary antibodies against phospho-SAPK/JNK (1:1,000), phospho-ERK (1:10,000), phospho-4E-BP (1:3,000), JNK (1:1,000), ERK (1:1,000), or α-tubulin (1:10,000) in TBST with 5% bovine serum albumin (BSA). Blots were then washed three times in TBST for 10 min each and further incubated with an HRP-linked second antibody in TBST with 1% BSA. Following three additional washes, immunoreactivity was visualized by chemiluminescence using Western Lightning Chemiluminescence Reagent *Plus* from PerkinElmer Life Sciences. Films exposed to the chemiluminescent reaction were scanned and quantified using an AlphaImager Imaging System and AlphaEaseFC software (Alpha Innotech, San Leandro, CA, USA).

### Kinase assay for JNK activity

To measure JNK activity, control or treated PGs (each of a pair of glands for control and treatment from 10 larvae) were lysed in JNK lysis buffer (20 mM Tris at pH 7.4, 150 mM NaCl, 1 mM EDTA, 1 mM EGTA, 1% Triton, 2.5 mM sodium pyrophosphate, 1 mM β-glycerolphosphate, 1 mM Na_3_VO_4_, and 1 μg/ml leupeptin). Subsequently, 200 μl of gland lysate was incubated with 20 μl of c-Jun fusion protein beads overnight at 4°C, and then a kinase assay was performed following the manufacturer's instructions (Cell Signaling Technology). The reaction was carried out for 30 min at 30°C and was stopped by boiling the samples in 3x SDS sample buffer. Levels of phospho-c-Jun were measured by Western blotting as described in the section “Western blot analysis”.

### Lambda protein phosphatase treatment

PG lysates were treated with lambda protein phosphatase (New England Biolabs, Beverly, MA, USA), which removes phosphates from serine, threonine, and tyrosine residues (Zhuo et al., 1993). The lysates were incubated for 30 min at 30°C with 1000 units of lambda protein phosphatase in 50 μl of reaction buffer according to the manufacturer's specifications. At the end of the incubation period, the sample was boiled for 10 min and stored at −70°C until the PAGE analysis.

### RNA extraction and quantitative real-time polymerase chain reaction (qRT-PCR)

Total RNA from PGs was extracted using the TRI Reagent (New England Biolabs, Beverly, MA, USA) according to the manufacturer's protocol. Purification of total RNA, reverse transcription and real-time qRT-PCR were performed as described previously (Young et al., 2012). Transcript levels were normalized to the *Bombyx* ribosomal protein 49 (rp49, GenBank accession: NM_001098282.1). The real-time qRT-PCR was performed using the following primers: *JNK* forward, 5′-ACCAAGACTGATATTGTGATAC-3′ and *JNK* reverse, 5′-AAGTTGTCCGTGTCCATA-3′; *Jun* forward, 5′-ATGACGAGCAATATCCTC-3′ and *Jun* reverse, 5′-GATCCGAGCTTCAACATT-3′; and rp49 forward, 5′-CAGGCGGTTCAAGGGTCAATAC-3′ and rp49 reverse, 5′-TGCTGGGCTCTTTCCACGA-3′.

### Data analysis

Data are shown as the mean ± standard error of the mean (SEM). Statistical comparisons between different groups were done using either Student's *t*-test for comparison of two groups or a one-way analysis of variance (ANOVA) followed by Tukey's test for more than two groups. A *p* < 0.05 was considered significant.

## Results

### *Bombyx mori* PGs expressed JNK, and PTTH stimulated JNK phosphorylation both *in vitro* and *in vivo*

Although there are 10 different splicing isoforms of the three JNK genes (JNK 1, 2, and 3) in mammals, only one JNK (DJNK) exists in *Drosophila*. *B. mori JNK* (*BmJNK*) was cloned as previously reported by Katsuma et al. (2007). BmJNK was respectively reported to have 79.8 and 74.5% amino acid sequence identities with *Drosophila* DJNK (Basket) and human JNK1 (Katsuma et al., 2007). In the kinase domain, *Bombyx* JNK contains all conserved residues of protein kinases, and the TPY motif between kinase subdomains VII and VIII, which is phosphorylated on threonine and tyrosine residues to activate JNKs (Riesgo-Escovar et al., 1996; Sluss et al., 1996; Davis, 2000). High similarities of *B. mori* JNK with those of human and *Drosophila* suggest that commercial antibodies against mammalian JNK can likely be used in *B. mori*.

**Figure 1:**
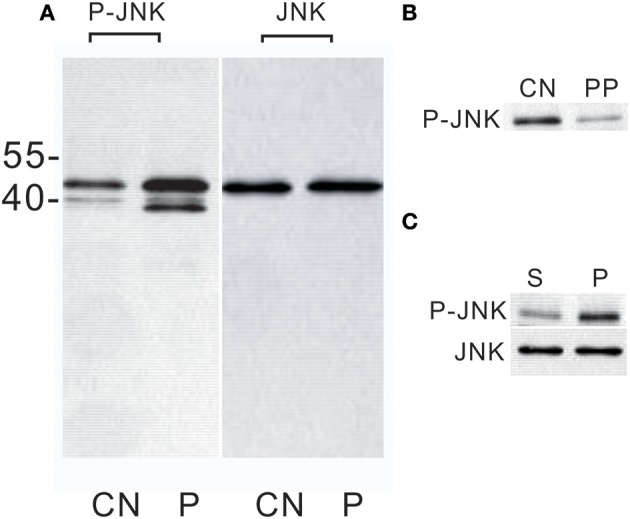
Western blot analysis of JNK phosphorylation in *Bombyx* PGs. **(A)** Effect of the PTTH *in vitro*. PGs were pre-incubated in control medium for 30 min and then transferred to control medium (CN) or medium containing the PTTH (P). PGs were incubated for 1 h. MW markers are shown on the left side of the gel. **(B)** Effect of lambda protein phosphatase treatment. CN, treated with buffer only; PP, treated with lambda protein phosphatase. **(C)** Effects of an *in vivo* PTTH injection on JNK phosphorylation. Larvae from day-6 last instar were injected with saline containing the PTTH (P) or saline only (S). At 30 min after the injection, PGs were quickly dissected out, and then immediately flash-frozen. Gland lysates were prepared and subjected to an immunoblot analysis with anti-phospho-JNK (P-JNK) and anti-JNK (JNK) antibodies. Results shown are representative of three independent experiments.

Previous studies showed that PTTH stimulates ERK phosphorylation in both *Manduca* and *Bombyx* PGs (Rybczynski et al., 2001; Lin and Gu, 2007). In the present study, we further examined whether PTTH stimulates JNK phosphorylation. Figure 1A shows whole blots of lysates of PGs. Using an anti-phospho-JNK (Thr183/Tyr185) antibody, one immunoreactive protein with a molecular weight (MW) of about 46 kDa was detected in the lysate of a control PG from day-6 last instar larvae. The PTTH greatly increased the phosphorylation level of this protein. In addition, the phosphorylation level of another immunoreactive protein with an MW of ~38 kDa was also strongly increased upon PTTH stimulation, although it remained low in the lysate of the control PG. Between these two bands, there was another small immunoreactive protein with an MW of ~40 kDa. Currently, it is not clear whether these two bands (of 38 and 40 kDa) correspond to minor splice variants of *Bombyx* JNK or were due to non-specificity of the antibody. Considering the difference in MW between *Bombyx* JNK and these two bands, we only focused on the 46 kDa *Bombyx* JNK in subsequent experiments.

Studies were conducted with an antibody that recognizes total JNK. Results (Figure 1A) showed that the JNK level did not change after PTTH treatment, thus confirming that PTTH activates phosphorylation levels of this kinase. Lysates of PTTH-stimulated PGs were incubated with lambda protein phosphatase prior to electrophoresis, and the immunoreactivity detected by the antibody directed against human anti-phospho-JNK decreased (Figure 1B); this indicates that the above antibody, raised against mammalian sequences, indeed recognized phosphorylated epitopes in *B. mori* PGs.

The above results clearly showed that the PTTH activated JNK phosphorylation of *B. mori* PGs *in vitro*. In subsequent experiments, we examined *in vivo* activation of JNK phosphorylation of PGs by PTTH. PTTH was injected into day-6 last instar larvae. After 30 min, the PGs were quickly dissected out, and JNK phosphorylation was examined and compared to that of control larvae. We chose 30 min after the *in vivo* PTTH injection because *in vitro* time-dependent effects of the PTTH revealed that the highest stimulation was detected 30 min after the PTTH injection (see below). Results showed that the PTTH injection greatly increased the JNK phosphorylation level, compared to that of the controls, verifying the *in vitro* stimulation of JNK phosphorylation (Figure 1C).

### PTTH stimulated JNK phosphorylation in time- and dose-dependent manners, and PTTH stimulation was tissue-specific

**Figure 2:**
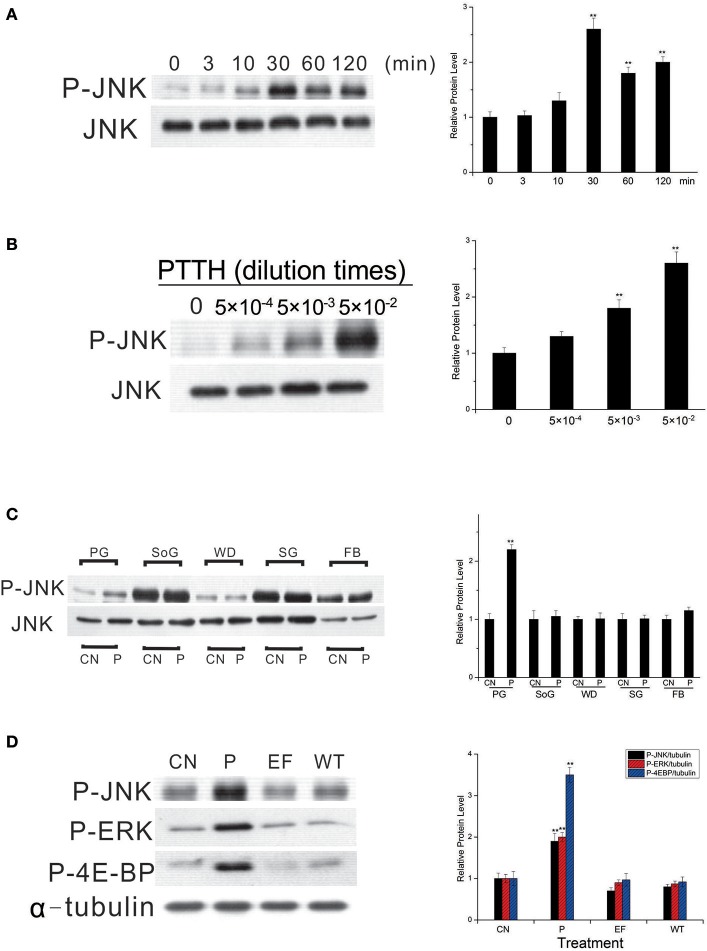
Time- **(A)** and dose- **(B)** dependent effects of JNK phosphorylation by the PTTH, tissue specificity of PTTH-stimulated JNK phosphorylation **(C)**, and the effect of treatment with extracellular fluid from cells only or cells infected with the wild-type (WT) AcMNPV on protein phosphorylation **(D)**. **(A,B)** Time- and dose-dependent effects. PGs were either treated with PTTH for the indicated time points **(A)**, treated with the indicated concentrations of PTTH, or incubated with control medium (0) for 60 min **(B)**. **(C)** Tissue specificity. PGs, subesophageal ganglia (SoG), wing disks (WD), salivary glands (SG), and fat body (FB) from day 6-last instar larvae were incubated with control medium (CN) or medium containing the PTTH (P) for 60 min. **(D)** Effect of treatment with either control medium (CN), the PTTH (P), extracellular fluid from cells only (EF), or cells infected with WT AcMNPV (WT) on the phosphorylation of JNK, ERK, and 4E-BP. Each lysate was prepared and subjected to an immunoblot analysis with anti-phospho-JNK (P-JNK), anti-phospho-ERK (P-ERK), anti-phospho-4E-BP (P-4E-BP), anti-JNK (JNK), and anti-α-tubulin (α-tubulin) antibodies. Results shown in the left panels are representative of three independent experiments. Data are expressed as multiples of change over the respective control after being normalized to the level of JNK (for **A–C**) or α-tubulin (for **D**). Asterisks indicate a significant difference compared to the respective control (by Student's *t*-test, ^**^p < 0.01).

In a subsequent experiment, we studied the *in vitro* JNK phosphorylation by the PTTH in greater detail. Figure 2A shows that a time-dependent increase in JNK phosphorylation *in vitro* was detected when PGs from day-6 last instar larvae were treated with the PTTH. Activation of JNK by the PTTH was detected after 10 min and reached a peak at 30 min after treatment, and activation persisted for 120 min (Figure 2A). Figure 2B shows the dose-dependent effects of the PTTH on JNK phosphorylation. To examine whether or not PTTH specifically exerts its action on *B. mori* PGs, PGs, subesophageal ganglions, wing disks, salivary glands, and a small piece of fat body were incubated with or without the PTTH. Results showed that only PGs, but not other tissues, exhibited activation of JNK phosphorylation after stimulation by the PTTH (Figure 2C), indicating the specificity of PTTH's action on PGs.

In addition, when PGs were treated with extracellular fluid from cells only or cells infected with WT AcMNPV, no activation of JNK phosphorylation was observed (O'Reilly et al., 1995); this indicates the specificity of the recombinant PTTH (Figure 2D). Treatment with the PTTH, but not with extracellular fluid from cells only or cells infected with the WT AcMNPV, stimulated ERK and 4E-BP phosphorylation, two well-documented signaling pathways downstream of PTTH stimulation (Gu et al., 2010, 2017), further confirming the specificity of the recombinant PTTH in the present study.

### Involvement of PLC and Ca^2+^ in PTTH-stimulated JNK phosphorylation

**Figure 3:**
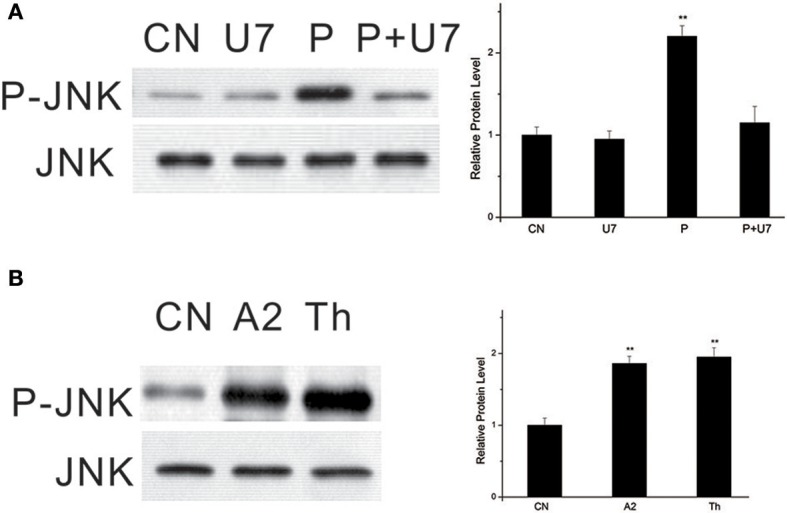
Effect of U73122 on PTTH-stimulated JNK phosphorylation **(A)** and stimulation of JNK phosphorylation by A23187 and thapsigargin **(B)**. **(A)** Effect of U73122. PGs were pretreated with either 10 μM U73122, or the vehicle alone for 30 min and transferred to medium containing the same dose of U73122, with or without the PTTH. CN, glands incubated in control medium; P, glands incubated in medium containing the PTTH only; U7: glands incubated in medium containing U73122 only; P+U7, glands incubated in medium containing both the PTTH and U73122. **(B)** Effects of A23187 and thapsigargin. Gland lysates were prepared and subjected to an immunoblot analysis with anti-phospho-JNK (P-JNK) and anti-JNK (JNK) antibodies. Results shown in the left panels are representative of three independent experiments. Data are expressed as multiples of change over the respective control after being normalized to the JNK level. Asterisks indicate a significant difference compared to the respective control (by Student's *t*-test, ^**^p < 0.01).

U73122, a selective pharmacological inhibitor of phosphoinositide-specific PLC (Smith et al., 1990), was used to demonstrate the involvement of PLC in PTTH-stimulated JNK phosphorylation. As shown in Figure 3A, pretreatment of PGs with U73122 (10 μM) blocked PTTH-stimulated JNK phosphorylation, thus indicating PLC's involvement.

To further examine whether PTTH-stimulated JNK phosphorylation is dependent on Ca^2+^, we used A23187, a Ca^2+^ ionophore. Figure 3B shows that treatment with A23187 greatly increased JNK phosphorylation. Similar to A23187, thapsigargin, an inhibitor of endoplasmic reticulum Ca^2+^-ATPase, also increased JNK phosphorylation. Gu et al. (2010) previously reported that A23187 and thapsigargin increase ERK phosphorylation and ecdysteroid secretion in *Bombyx* PGs.

### Effects of inhibitors of MEK and PI3K on PTTH-stimulated JNK phosphorylation

**Figure 4:**
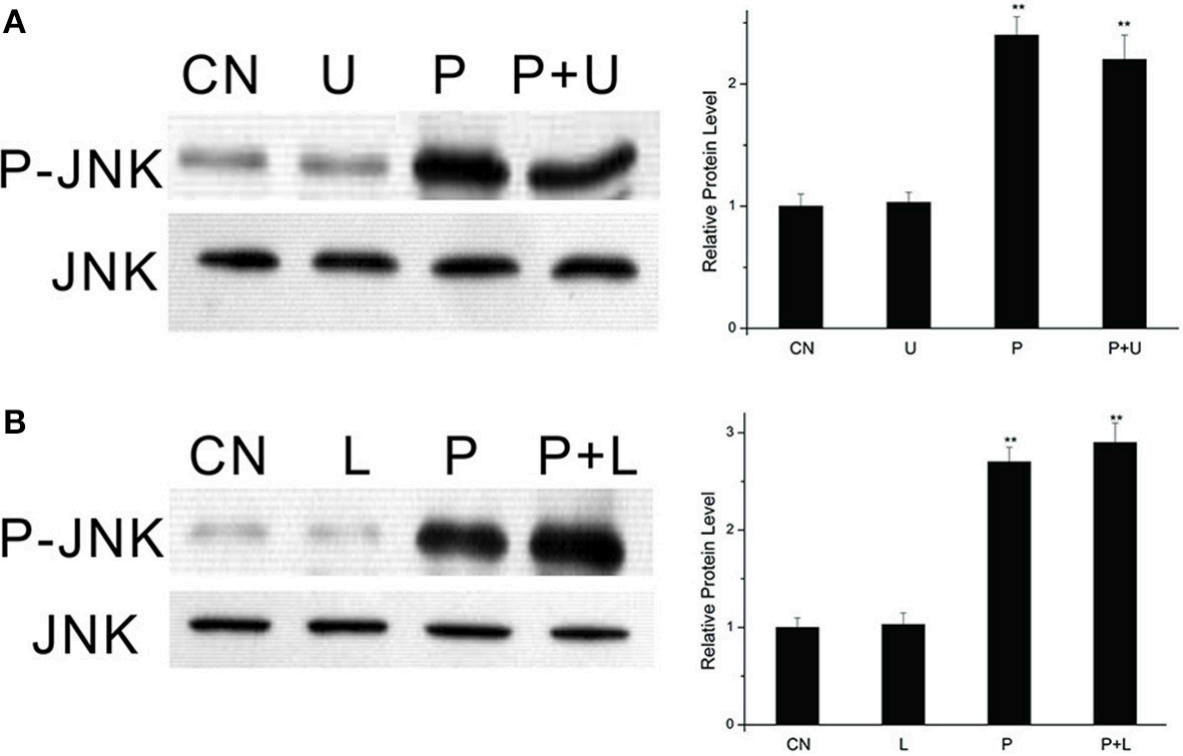
Effects of U0126 **(A)** and LY294002 **(B)** on PTTH-stimulated JNK phosphorylation. PGs were pretreated with either 10 μM U0126, 50 μM LY294002, or control medium for 30 min, and then transferred to medium containing the same dose of each inhibitor, with or without the PTTH. CN, PGs incubated in control medium; P, PGs incubated in medium containing the PTTH only; U, PGs incubated in medium containing U0126 only; P+U, PGs incubated in medium containing both the PTTH and U0126; L, PGs incubated in medium containing LY294002 only; P+L, PGs incubated in medium containing both the PTTH and LY294002. The incubation time was 60 min. Gland lysates were prepared and subjected to an immunoblot analysis with anti-phospho-JNK (P-JNK) and anti-JNK (JNK) antibodies. Results shown in the left panels are representative of three independent experiments. Data are expressed as multiples of change over the respective control after being normalized to the level of JNK. Asterisks indicate a significant difference compared to the respective control (by Student's *t*-test, ^**^p < 0.01).

We used U0126, a specific MEK inhibitor, to determine whether PTTH-stimulated JNK phosphorylation is linked to ERK phosphorylation. As shown in Figure 4A, U0126 did not block PTTH-stimulated JNK phosphorylation. We further examined the effect of LY294002, a PI3K inhibitor, on PTTH-stimulated JNK phosphorylation. PGs pretreated with LY294002 were subsequently challenged with the PTTH. Figure 4B shows that LY294002 did not affect PTTH-stimulated JNK phosphorylation, which indicates that PI3K signaling is not related in PTTH-stimulated JNK phosphorylation.

### Activation of JNK signaling by the PTTH is dependent on ROS

**Figure 5:**
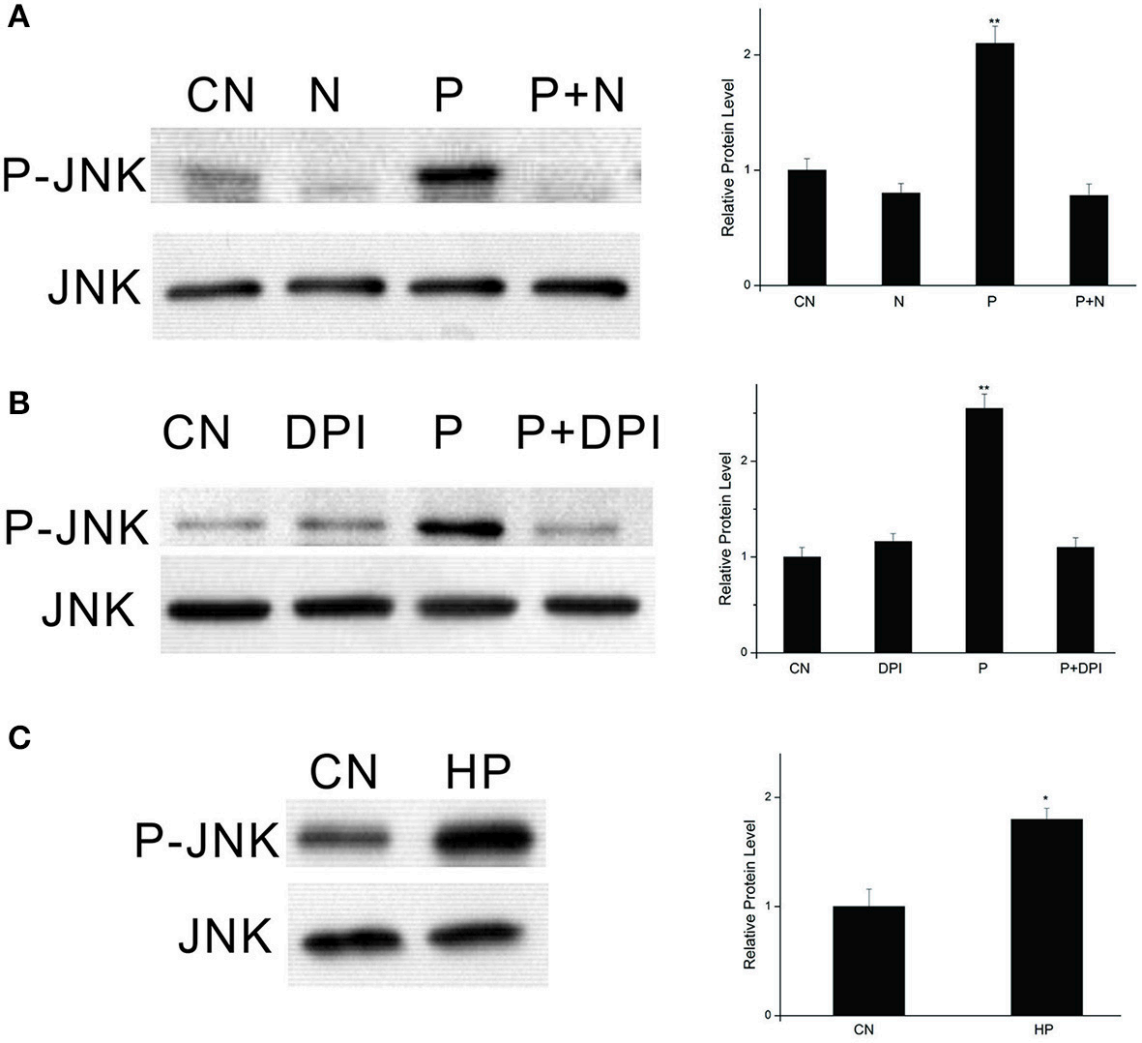
Effects of NAC **(A)** and DPI **(B)** on PTTH-stimulated JNK phosphorylation and effect of exogenous H_2_O_2_ on JNK phosphorylation **(C)**. PGs were pretreated with either NAC (20 mM), DPI (10 μM), or vehicle alone for 30 min, and then transferred to medium containing the same dose of the inhibitors, with or without the PTTH. Incubation was maintained for 60 min. CN, PGs incubated in control medium; N, PGs incubated in medium containing NAC only; P, PGs incubated in medium containing the PTTH only; P+N, PGs incubated in medium containing both the PTTH and NAC; DPI, PGs incubated in medium containing DPI only; P+DPI, PGs incubated in medium containing both the PTTH and DPI; HP, PGs treated with H_2_O_2_ (1 mM). Gland lysates were prepared and subjected to an immunoblot analysis with anti-phospho-JNK (P-JNK) and anti-JNK (JNK) antibodies. Results shown in the left panels are representative of three independent experiments. Data are expressed as multiples of change over the respective control after being normalized to the JNK level. Asterisks indicate a significant difference compared to the respective control (by Student's *t*-test, ^*^p < 0.05; ^**^p < 0.01).

In our previous study, we found that PTTH-stimulated phosphorylation of ERK is dependent on the redox status (Hsieh et al., 2014). To determine whether the JNK signaling pathway is redox-sensitive, PGs were pretreated with NAC, a superoxide scavenger, to block PTTH-stimulated ROS production (Hsieh et al., 2013), and then were stimulated with the PTTH. As shown in Figure 5A, blocking the PTTH-stimulated production of ROS by NAC completely blocked PTTH-stimulated JNK phosphorylation compared to PTTH treatment only. Treatment with a mitochondrial oxidative phosphorylation inhibitor (DPI) also greatly decreased PTTH-stimulated JNK phosphorylation (Figure 5B). In addition, treatment with NAC also reduced the basal JNK phosphorylation level compared to the controls, possibly due to inhibition of ROS production. Figure 5C shows that treatment with 1 mM of H_2_O_2_ greatly stimulated JNK phosphorylation. This result demonstrated that ROS alone can regulate JNK phosphorylation, thus confirming that ROS play a critical role in PTTH-stimulated JNK signaling. These findings suggest that JNK phosphorylation might be a critical component of the redox-sensitive signaling pathway activated by the PTTH in *B. mori* PGs.

### Effects of SP600125 on PTTH-stimulated JNK phosphorylation and ecdysteroid synthesis

**Figure 6:**
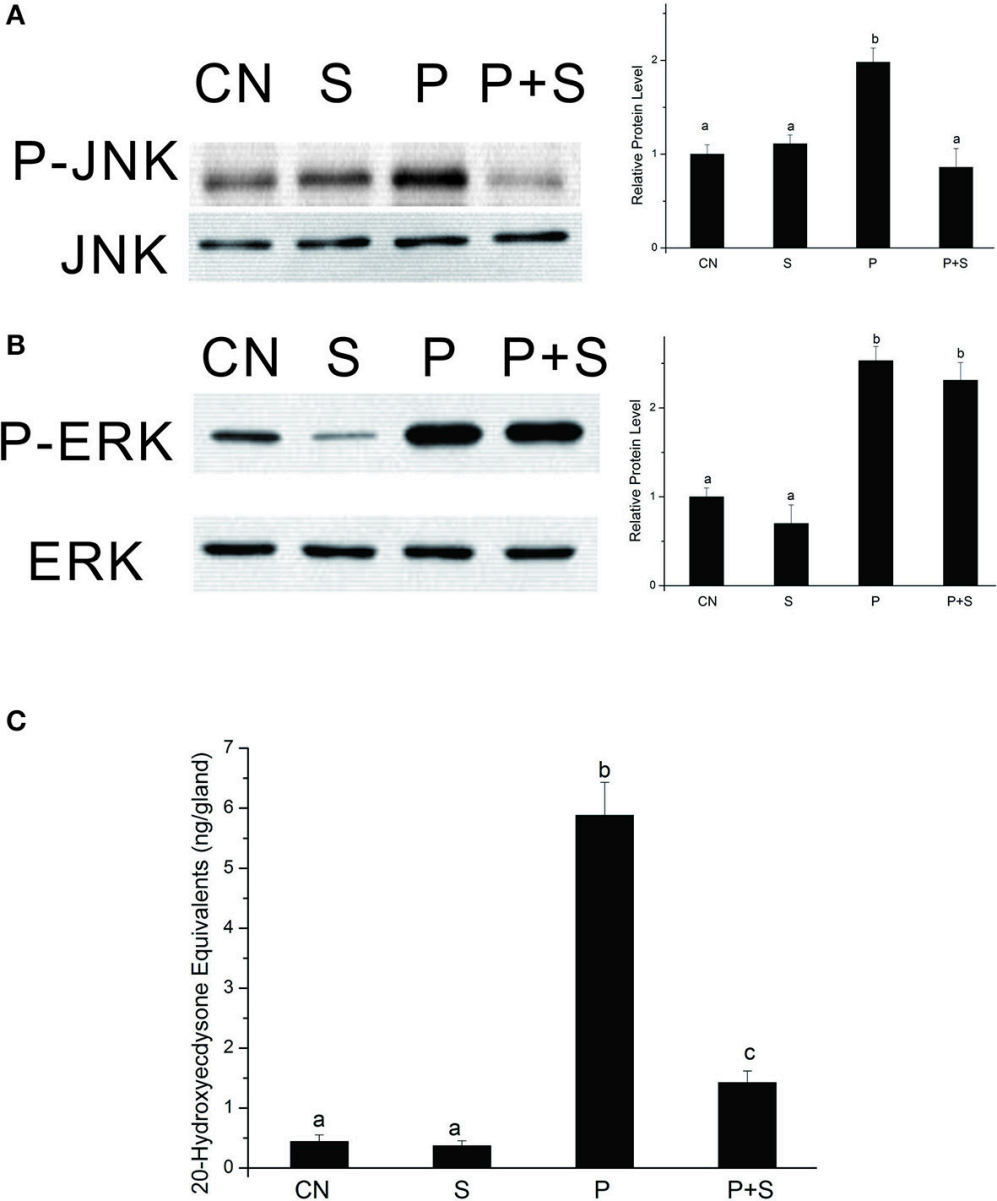
Effects of SP600125 on PTTH-stimulated phosphorylation of JNK **(A)** and ERK **(B)** and ecdysteroid secretion **(C)**. PGs were pretreated with 20 μM SP600125 or vehicle alone for 30 min, and then transferred to medium containing the same dose of SP600125, with or without the PTTH. Incubation was maintained for 60 min. Gland lysates were prepared and subjected to an immunoblot analysis with anti-phospho-JNK (P-JNK), anti-JNK (JNK), anti-phospho-ERK (P-ERK), and anti-ERK (ERK) antibodies. CN, glands incubated in control medium; S, glands incubated in medium containing 20 μM SP600125 only; P, glands incubated in medium containing the PTTH only; P+S, glands incubated in medium containing both the PTTH and 20 μM SP600125. Results shown in the left panels (for **A,B**) are representative of three independent experiments. Data are expressed as multiples of change over the respective control after being normalized to the level of JNK (for **A**) or ERK (for **B**). Ecdysteroid released into the medium was determined by an RIA. Different letters above the bars indicate a significant difference (ANOVA followed by Tukey's multiple-comparisons test, *p* < 0.05).

The above data clearly show that the PTTH stimulates JNK phosphorylation. We further confirmed the role of JNK signaling in regulating PTTH-stimulated ecdysteroid synthesis with SP600125, a specific JNK kinase inhibitor. After PGs were pretreated with SP600125, they were challenged with the PTTH. Phosphorylated JNK and ERK levels in PGs were examined, and ecdysteroid secretion was determined. Results (Figure 6) showed that SP600125 treatment decreased PTTH-stimulated JNK phosphorylation levels, but did not affect PTTH-stimulated ERK phosphorylation levels. The basal ERK phosphorylation level was inhibited by treatment with SP600125, indicating that basal ERK phosphorylation is sensitive to this inhibitor. Moreover, although SP600125 treatment did not completely block PTTH-stimulated ecdysteroid secretion, it greatly inhibited it. These results confirm the involvement of JNK signaling in PTTH-stimulated ecdysteroid secretion.

### Effect of the PTTH on the kinase activity of JNK

**Figure 7:**
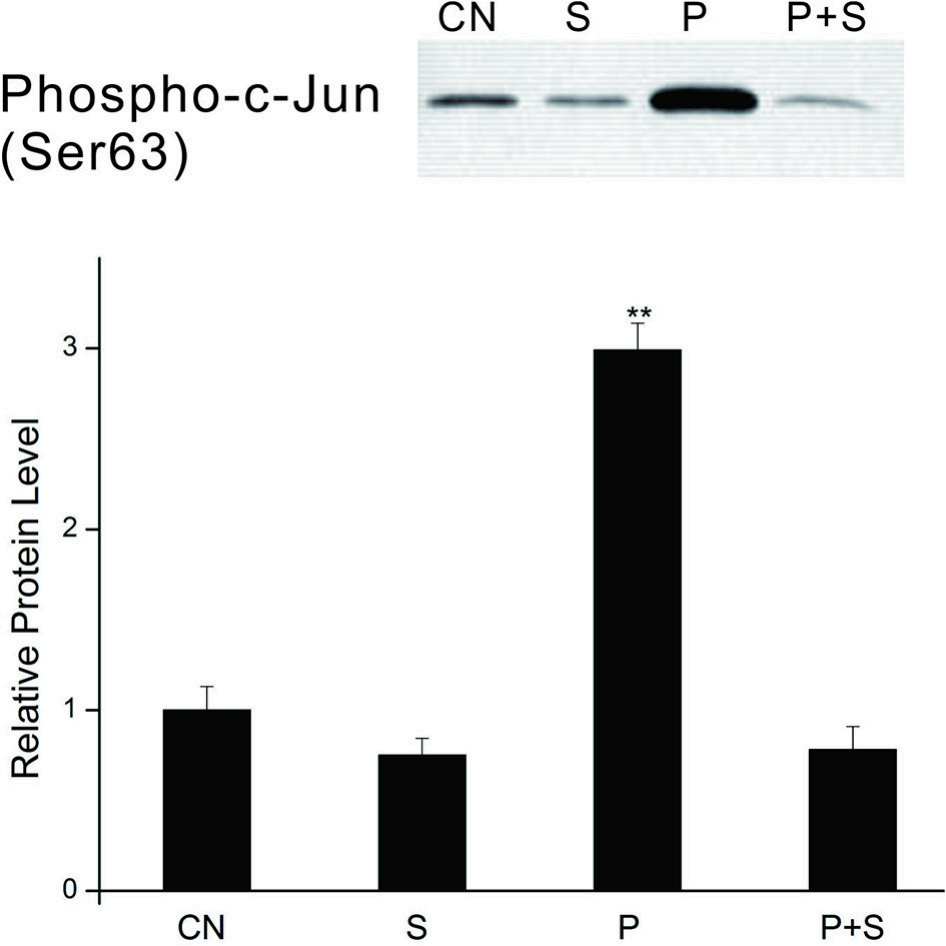
Effects of SP600125 on the PTTH-stimulated kinase activity of JNK. PGs were pretreated with 20 μM SP 600125 or vehicle alone for 30 min, and then transferred to medium containing the same dose of SP600125, with or without the PTTH. Incubation was maintained for 60 min. CN, glands incubated in control medium; S, glands incubated in medium containing 20 μM SP600125 only; P, glands incubated in medium containing the PTTH only; P+S, glands incubated in medium containing both the PTTH and 20 μM SP600125. *In vitro* kinase assays using c-Jun fusion protein beads were performed on gland lysates and subsequently analyzed by immunoblotting with an antibody to phospho-c-Jun. Results shown in the top panels are representative of three independent experiments. Data are expressed as multiples of change over the control. Asterisks indicate a significant difference compared to the control (by Student's *t*-test, ^**^p < 0.01).

We further examined the effect of the PTTH on the kinase activity of JNK. Lysates from PGs were incubated with 20 μl of c-Jun fusion protein beads overnight, and then a kinase assay was performed per the manufacturer's instructions. Results (Figure 7) show that the kinase activity of JNK from PTTH-stimulated PG lysates greatly increased compared to that from control gland lysates, and that SP600125 inhibited the PTTH-stimulated kinase activity of JNK. In addition, SP600125 also inhibited the basal kinase activity of JNK. These results confirmed that the PTTH-activated phosphorylation of JNK is indeed related to its kinase activity.

### Effect of the PTTH on JNK and jun gene expressions

**Figure 8:**
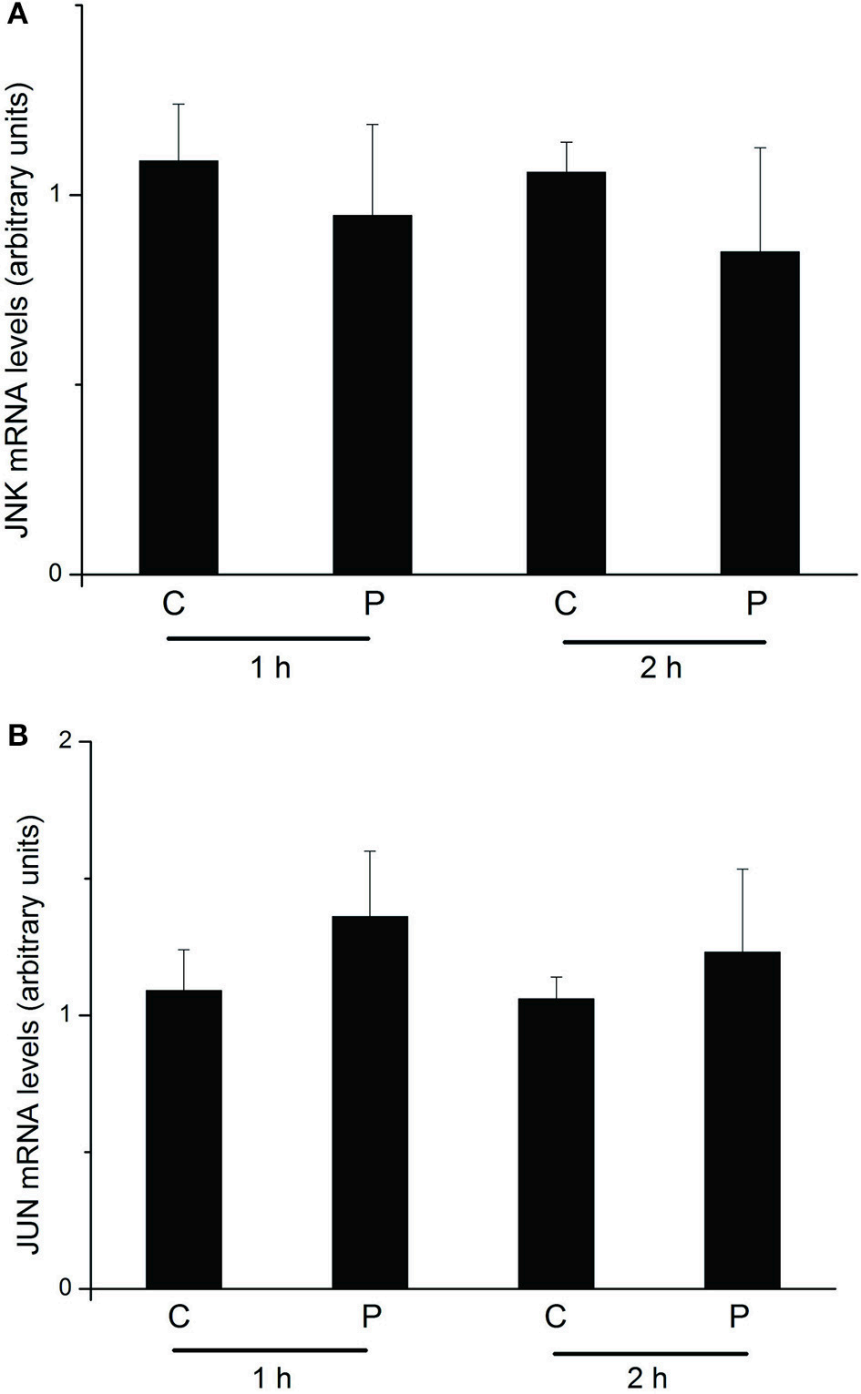
Changes in mRNA expression levels of *JNK* **(A)** and *Jun* **(B)** upon treatment with the PTTH. PGs were preincubated in medium for 30 min and then transferred to medium containing the PTTH (P) or control medium (C). Incubation was maintained for 1 and 2 h. After each incubation, total RNA was extracted from PGs, and mRNA expression levels of JNK and Jun were determined by an RT-qPCR. Each bar represents the mean ± SEM of four separate assays.

We further conducted an experiment to determine changes in *JNK* (GenBank accession: NP_001103396.1) and *Jun* (http://silkworm.genomics.org.cn/: BGIBMGA004164) mRNA expression levels upon PTTH treatment. As shown in Figure 8, no significant differences were detected in *JNK* or *Jun* transcription levels between control glands and those treated with the PTTH during the 1- or 2-h incubation periods after *in vitro* treatment with PTTH.

## Discussion

The pathways that mediate ecdysteroidogenesis in response to PTTH stimulation are complex and not fully understood at the molecular level. Several studies demonstrated the involvement of the PKA, PKC, ERK, and AMPK/TOR signaling pathways (Smith and Rybczynski, 2012; Gu et al., 2017). In the present study, we demonstrated that PTTH stimulates phosphorylation of JNK in *B. mori* PGs. *In vitro* activation by the PTTH of JNK phosphorylation in *B. mori* PGs was also confirmed by *in vivo* experiments: a PTTH injection into day-6 last instar larvae greatly augmented JNK phosphorylation of PGs. The kinase assay of JNK in PGs confirmed its stimulation by the PTTH. However, the PTTH did not affect *JNK* or *Jun* mRNA expressions. Although ERK/MAPK signaling was previously demonstrated to be involved in PTTH-stimulated ecdysteroidogenesis in PGs of both *M. sexta* (Rybczynski et al., 2001) and *B. mori* (Lin and Gu, 2007; Gu et al., 2010), this is the first study, to our knowledge, to demonstrate JNK phosphorylation by the PTTH both *in vitro* and *in vivo*.

**Figure 9:**
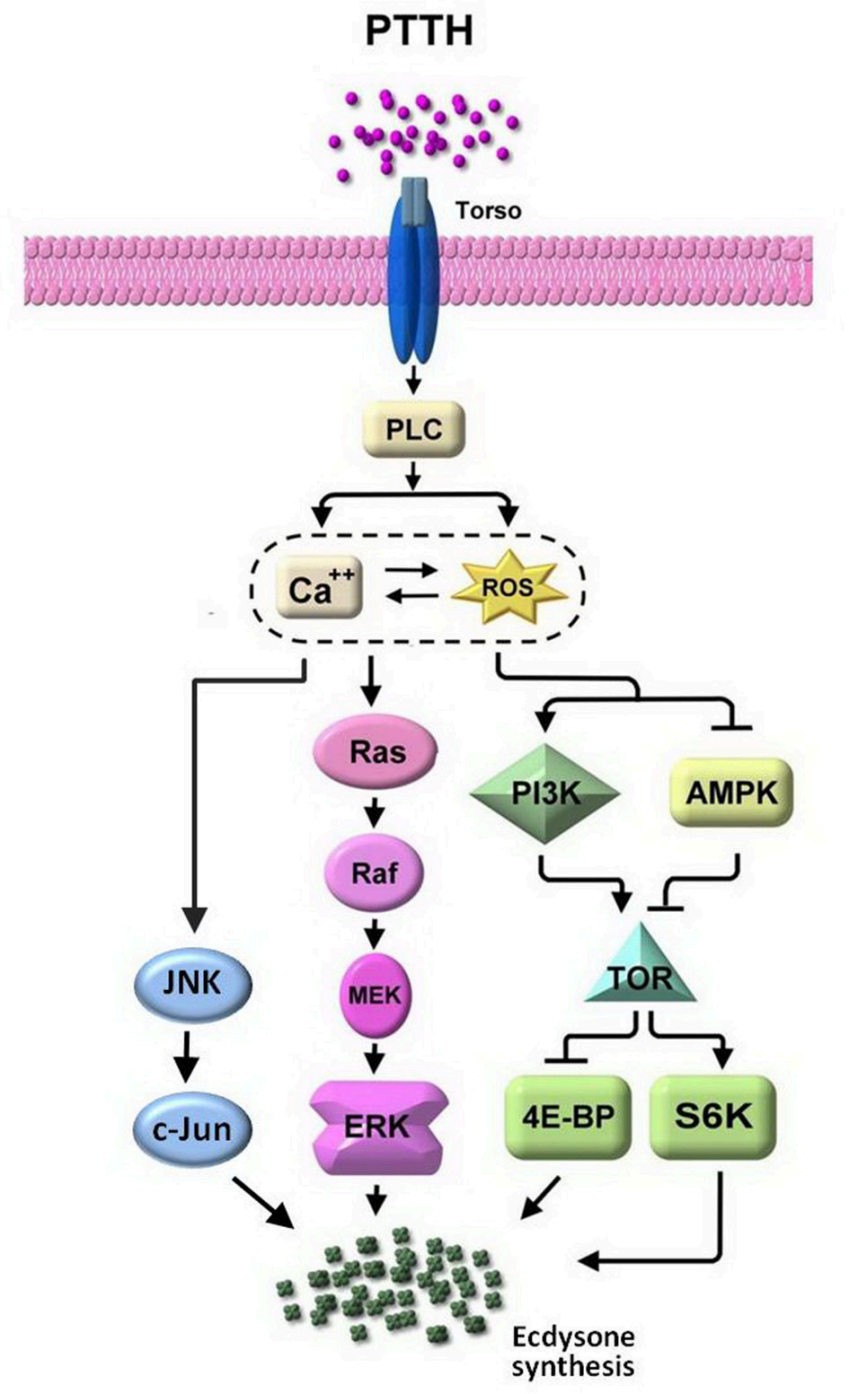
The signaling network involved in PTTH-stimulated ecdysteroidogenesis in *Bombyx* PGs. See text for details.

We further showed that PTTH-stimulated ecdysteroidogenesis in *B. mori* PGs was suppressed by SP600125, a specific inhibitor of JNK (Bennett et al., 2001). SP600125 was found to truly attenuate JNK phosphorylation induced by the PTTH in PGs. A kinase assay of JNK confirmed its inhibitory effect. ERK was previously reported to be involved in phosphorylation induced by the PTTH (Rybczynski et al., 2001; Lin and Gu, 2007). However, SP600125 did not affect PTTH-stimulated ERK phosphorylation. Therefore, SP600125-induced inhibition of PTTH-stimulated ecdysteroid synthesis is due to inhibition of JNK phosphorylation. Based on these findings, it is most likely that ecdysteroidogenesis stimulated by the PTTH is mediated through phosphorylation of both ERK and JNK in *B. mori* PGs (Figure 9). However, it should be stated that PTTH-mediated signaling transduction pathways are far more complex than illustrated in Figure 9, and that many downstream effectors are still being explored. It is suggested that PTTH binding alters the protein conformation of Torso such that it stimulates nearby effector proteins which catalyze the production of second messengers, and finally propagate the signal through various intricate signaling pathways to induce new gene expressions. In addition, our previous study demonstrated that TOR signaling is involved in PTTH-stimulated ecdysteroidogenesis in *Bombyx* PGs (Gu et al., 2012). PGs may integrate signaling from the PTTH, insulin, nutrients, or other factors to regulate the AMPK/TOR signaling pathway in order to modulate cell growth and ecdysteroidogenesis (Gu et al., 2012, 2015; Smith et al., 2014).

The MAPK superfamily was found to mediate intracellular signaling of extracellular agonists and play important roles in various cellular functions including proliferation, differentiation, and apoptosis in a variety of cells (Roux and Blenis, 2004). Conventional MAPK proteins consist of three family members (Johnson and Lapadat, 2002): ERK, JNK, and p38. ERK is mainly activated by mitogens and differentiation signals, while JNK and p38 are activated by stress stimuli and many different factors, including EGF, PDGF, transforming growth factor (TGF)-β, and tumor necrosis factor (Roux and Blenis, 2004). In the present study, we found that pretreatment with neither U0126, a MEK inhibitor, nor LY294002, a PI3K inhibitor, greatly inhibited JNK phosphorylation stimulated by the PTTH. This result suggests that PTTH-stimulated JNK signaling is independent of both ERK and PI3K. The independence of PTTH-stimulated ERK signaling from PI3K was previously demonstrated (Gu et al., 2010). In addition, we detected increased JNK phosphorylation after PGs were treated with agents that directly elevated the intracellular Ca^2+^ concentration (either A23187 or thapsigargin). U73122 was also found to block PTTH-stimulated JNK phosphorylation. These results clearly indicate that PTTH-stimulated JNK signaling is both PLC- and /Ca^2+^-dependent.

Our previous study demonstrated that mitochondrion-derived ROS signaling and Ca^2+^ signaling are together involved in PTTH-stimulated ecdysteroidogenesis (Hsieh et al., 2013). We further found that ROS signaling lies upstream of ERK, 4E-BP, and AMPK phosphorylation (Hsieh et al., 2014). In the present study, we investigated the regulatory function of the redox state on JNK phosphorylation. Our results indicate that pretreatment with either an antioxidant (NAC) or DPI blocked PTTH-regulated JNK phosphorylation. In addition, we found that ROS alone could activate JNK phosphorylation. These results indicate that similar to ERK signaling, PTTH-stimulated JNK phosphorylation is a redox-regulated signaling pathway. Although redox regulation of MAPK signaling is well-documented in mammalian systems (Son et al., 2013), this, to our knowledge, is the first study to demonstrate redox regulation of JNK signaling in the endocrine system of an insect. However, it is necessary to view this conclusion with caution, because the specificities of the experimental inhibitors we used have not been proven in insects.

In larval insects, the PTTH is the major stimulator of ecdysteroid secretion in PGs. Early studies showed that the three-dimensional structure of the PTTH resembles those of mammalian growth factors including PDGF, NGF, and TGF-β (Noguti et al., 1995). Although the present study clearly showed that JNK activation is involved in PTTH-stimulated ecdysteroid secretion by *B. mori* PGs during a short-term incubation period, it is not clear whether or not PTTH-stimulated JNK signaling has additional roles. In mammalian cells, it was revealed that growth factors and oncogenes induced sustained activation of JNK to mediate their effects on survival, proliferation, and transformation (Davis, 2000). Activation of JNK in Rat-1 fibroblasts was shown to contribute to insulin-stimulated transcription of genes regulated by activating protein-1 (Miller et al., 1996). Furthermore, JNK is important for AP-1-mediated transcriptional activation and cell growth in response to insulin-like growth factor-1 in IEC-6 intestinal cells (Simmons et al., 1995). Like ERK, JNK may be re-localized from the cytoplasm to nuclei following stimulation (Roux and Blenis, 2004; Krishna and Narang, 2008). A wide range of nuclear proteins, predominantly transcription factors and nuclear hormone receptors, were found to be JNK substrates (Weston and Davis, 2002). These directly affect its gene expression. A well-known substrate for JNK is the transcription factor, c-Jun. Phosphorylation of c-Jun by JNK leads to increased c-Jun-dependent transcription (Weston and Davis, 2002). In the present study, phosphorylation of c-Jun by the PTTH was confirmed. Thus, it is possible that PTTH-stimulated JNK phosphorylation may play a role in regulating activation of transcription factors related to c-Jun, leading to a sustained increase in ecdysteroidogenesis. Further study is needed to clarify downstream signaling pathways of JNK phosphorylation stimulated by the PTTH.

In summary, our study demonstrates that JNK represents another class of signaling molecules which reside downstream of the PTTH receptor and which may be necessary for ecdysteroid biosynthesis. Understanding the roles of JNK in signaling and identifying mechanisms that regulate JNK activation in response to PTTH and its downstream signaling pathways are fundamental to a clearer understanding of signaling events that regulate ecdysteroidogenesis in insect PGs.

## Author contributions

S-HG: designed and conducted the experiments, and wrote the manuscript; GL and SL: provided some ideas and contributed important resources, techniques, and reagents; H-YH and P-LL performed the experiments.

### Conflict of interest statement

The authors declare that the research was conducted in the absence of any commercial or financial relationships that could be construed as a potential conflict of interest.
